# 자연주의 출산한 여성의 출산실태와 회음부 손상: 일개 자연주의 출산 병원 의무기록을 중심으로

**DOI:** 10.4069/kjwhn.2021.08.31

**Published:** 2021-12-24

**Authors:** Kyung Won Kim, Sunhee Lee

**Affiliations:** 1 Department of Nursing, Daegu Haany University, Daegu, Korea; 1 대구한의대학교 간호학과; 2 Department of Nursing, Gimcheon University, Gimcheon, Korea; 2 김천대학교 간호학과

**Keywords:** Lacerations, Natural childbirth, Parity, Perineum, Pregnancy, 열상, 자연주의 출산, 출산력, 회음, 임신

## Abstract

**Purpose:**

This study aimed to determine the actual state of childbirth in Korean women with natural childbirth and the degree of damage to the perineum during childbirth.

**Methods:**

This retrospective study analyzed the medical records of mothers who had natural childbirth at a hospital in Seoul, Korea in 2018. Data from 358 women with cephalic births at greater than 37 gestational weeks were analyzed. To determine natural childbirth characteristics and the degree of damage to the perineum, descriptive statistics were done. The difference in the degree of perineal injury according to obstetric characteristics was analyzed using independent t-test and one-way analysis of variance.

**Results:**

The mean age was 33.18±3.68 years, and 49.2% were primiparas, while 39% gave birth with a doula. The degree of perineal damage differed by age (F=9.15, *p*<.001), parity (t=19.13, *p*<.001), number of births in multiparity (F=3.68, *p*=.027), previous vaginal delivery in multiparity (F=3.00, *p*=.032) and birthing posture (F=7.44, *p*<.001). Having received therapeutic procedures (t=–4.62, *p*<.001), specifically fluid administration (t=–2.72, *p*=.007), oxygen supply (t=-–2.76, *p*=.006) and epidural anesthesia (t=–2.77, *p*=.006) were statistically significant for perineal damage. There were no differences, however, by gestational period, doula use, water room use in labor, baby head circumference, or birth weight.

**Conclusion:**

Study findings suggest that support for older women, primiparas, and those who require therapeutic procedures may help to decrease the possibility of perineal damage during childbirth. As perineal damage was also associated with birthing posture, this should be considered when providing intrapartum nursing care.

## Introduction

조산원에서 이루어지던 많은 수의 자연분만(vaginal delivery)이 병원 분만으로 옮겨가면서 우리의 출산문화는 변화를 맞게 되었다[1]. 전국민 의료보험을 시작으로 국내 자연분만의 99.5%가 병원에서 이루어지며[2], 여성들은 조산사가 가진 출산지식과 기술보다는 첨단 의료장비가 있고 감염 관리가 잘되는 병원에서의 출산을 선호하여 왔다[3]. 여기에는 병원 출산에 무통분만이 도입되고 고위험 임신과 출산 시 응급상황에 상대적으로 잘 대처할 수 있어 안전하게 출산을 할 수 있다는 이유도 작용을 하였다.

실제 여성들은 첨단 의료장비로 산부와 태아의 건강을 살피면서 안전하게 출산할 수 있다는 점에서 병원 자연분만을 선택하였지만 병원 의료서비스에 대한 만족도는 조산원보다 낮은 것으로 나타났다[4]. 더욱이, 여성들은 출산과정에서 겪은 비인격적인 대우와 과도한 의료 처치로 위축되면서 의료인이 주도하는 분만을 따라가기보다는 스스로가 몸의 주체가 되어 출산에 참여하는 자기 주도적인 출산[5]과 의료적 처치가 개입되지 않는 자연주의 출산(natural childbirth) 방법[6]을 찾게 되었다.

자연주의 출산은 마취, 진정, 수술 등을 배제하고 자연스럽게 질을 통해 아기를 낳는 것으로[7], 의사와 조산사가 있는 공간에서 산부 스스로 진통과 출산을 주도하거나 둘라(doula) 교육을 받은 출산전문가의 도움을 받는다[8]. 국외 자연주의 출산연구를 보면 회음절개술을 선택적 시술로 바꾸고 다양한 비약물적 통증조절법을 적용하니 산부의 불안도가 낮아지고 출산 만족도와 성취감이 높았으며[9] 자연주의 출산에 둘라 지지가 병행되면서 통증과 출산시간이 감소되었다고 한다[10]. 그러나 국내의 경우 자연주의 출산을 하는 기관도 많지 않은 데다 둘라 지지 프로그램 개발[8]과 아버지의 둘라 참여[11], 수중실을 사용한 출산[12]에 관한 연구만 있을 뿐 자연주의 출산의 분만 진행과정과 그 성과를 파악한 연구는 부족한 실정이다. 덧붙여 안전한 출산과 응급상황에 대처한다는 명목으로 시행되던 회음절개술, 삭모, 관장이 비인간적인 처치로 평가되고 있다[13]. 회음절개술의 경우 회음부 손상을 최소화하면서 질 입구의 직경을 늘려 출산 진행을 촉진한다는 취지로 시행하여 왔으나[14], 이로 인해 산모는 출혈과 감염에 노출되며 절개방법과 시술자에 따라서 3–4도 회음 열상을 입는 경우가 많고 산모에게 요실금은 물론 직장과 질의 누공이 발생하는 등[15-17]의 문제가 있어 의료적 개입이 없는 자연주의 출산방식에 대한 관심이 고조되고 있다.

회음절개술을 적용하지 않는 자연주의 출산에서도 회음부 손상이 92.7%이지만, 대부분 1–2도의 열상인 점을 감안한다면[12] 자연주의 출산 산부의 출산 실태를 통해 회음부 손상 정도를 파악해보는 것도 필요하다. 초산, 기구분만, 신생아의 체중이 회음부 손상을 유발하며[17] 산부의 연령, 과숙임신, 유도분만, 분만2기 지연, 경막외 마취가 회음손상의 위험인자임[17-19]을 보고한 연구결과를 확인하여 향후 회음손상을 줄이는 방안을 모색할 수 있을 것이다.

이에 본 연구에서는 자연주의 출산을 한 여성들의 출산 실태를 파악하여 자연주의 출산방법과 그 내용을 확인하고자 하였다. 덧붙여, 회음절개술을 적용하지 않은 자연주의 출산에서 회음부 손상 정도와 그 관련 요인들도 확인하여 자연주의 출산을 하는 여성들을 위한 기초자료로 제공하고자 한다.

본 연구의 목적은 자연주의 출산 여성의 출산 실태와 출산과정에서 발생하는 회음부 손상 정도를 파악하는 것이다. 구체적인 목적은 다음과 같다.

1) 자연주의 출산 여성의 출산 실태와 회음부 손상 정도를 확인한다.

2) 자연주의 출산 여성의 산과적 특성에 따른 회음부 손상 정도의 차이를 확인한다.

## Methods

Ethics statement: This study was approved by the Institutional Review Board (IRB) of Gimcheon University (GU-201905-HRa-05-02-9), and obtaining informed consent was exempted by the IRB because data collection was done from chart reviews; there was no sensitive information and the data were anonymous.

### 연구 설계

본 연구는 자연주의 출산을 하는 병원의 의무기록을 분석하여 출산 실태와 회음부 손상 정도를 파악하기 위한 후향적 조사연구이다.

### 연구 대상

연구 대상자는 국내에서 자연주의 출산을 시도하는 의료기관 중 본 연구의 목적을 이해하고 동의한 서울 소재 일개 산부인과 병원에서 후향적으로 선정하였다. 본 기관은 자연주의 출산을 위해 간호조산사와 자격을 갖춘 둘라가 함께 출산을 수행하고 있었다. 2018년도 1월 1일에서 12월 31일까지의 기간 중 37주 이상, 두정위로 자연주의 출산을 한 397명의 여성 중 34명(외국인, 조기 출산, 유도분만, 미성년자, 그리고 둔위 출산)을 제외한 363명을 일차 선별하였다. 연구자는 일차 선별한 대상자의 의무기록을 검토하여 내용이 불충분한 5명의 의무기록을 제외하고 총 358명 여성 대상자의 의무기록을 분석하였다. 본 대상자들의 의무기록은 단일기관 전체 대상을 표본으로 하여 표본편향은 없었다.

### 연구 도구

자연주의 출산 여성의 실태에 관한 도구는 의무기록을 중심으로 도구를 선정하였다. 일반적 특성으로는 연령(세), 체중 증가(<11.5 kg, 부족; 11.5–16.0 kg, 적정; >16 kg, 과잉), 임신 전 체질량지수(body mass index, BMI), 출산 시 BMI [20]이며 BMI는 <18.5 kg/m^2^가 저체중, 18.5–22.9 kg/m^2^를 정상, 23–24.9 kg/m^2^를 과체중, ≥25 kg/m^2^를 비만으로 분류하였다[21].

산과적 특성으로는 출산력(초산부, 경산부), 경산부의 출산횟수(1–6회), 재태기간(주), 경산부에서 이전 질식분만(횟수), 경산부에서 이전 제왕절개 분만(유무), 둘라와 출산 (유무), 분만1기에서의 수중실 사용(유무), 출산 동반자(남편, 친정모, 형제자매, 기타), 회음부 손상 정도(회음절개술 유무에 따라 손상 없음; 경증, 1–2도 열상; 중증, 3-4도 열상), 아기 출산 시의 자세(반좌위, 수중에 앉아서, 옆으로 누워서, 앉아서, 엎드려서), 분만 1-3기 소요시간(분), 치료적 처치(유무), 치료적 처치 종류(항생제 투여, 수액 투여, 산소 공급, 경막외 마취, 인공 양막 파열), 아기 성별(남, 여), 아기 머리둘레(cm), 아기 출생 시 체중(g), 1분과 5분의 아프가 점수(Apgar score) 등이었다.

### 자료 수집

본 연구자는 연구 실시 기관을 방문하여 일개 의무기록을 검토하고 연구 실시기관에서 제공해줄 수 있는 항목을 논의 후 항목을 체계적이며 통일된 방식으로 검토할 수 있도록 코딩북을 만들어 연구보조원 1명을 교육하였다. 연구보조원은 연구 실시 기관에 근무하는 조산사이며 석사 학위 소지자로 의무기록 검토 및 자료입력 방법에 대해 잘 알고 있었으며, 연구자료는 개인정보를 식별할 수 없도록 일련의 번호를 부여하여 제공받았다. 연구보조원은 2019년 7월 1일에서 7월 31일까지 대상자의 의무기록 내용을 코딩 북에 자료 입력하였다. 연구에 필요한 대상자의 일반적, 산과적 특성 및 출산 관련 자료는 연구 실시 기관 원장의 동의를 거쳐 대상자의 개인정보가 식별되지 않는 범위 내에서 제공받았다.

### 자료 분석

자료는 IBM SPSS Statistics ver. 28.0 for Windows (IBM Corp., Armonk, NY, USA)를 이용하여 출산 실태에 관한 자료를 빈도, 백분율, 평균, 표준편차로 분석하였다. 일반적 및 산과적 특성에 따른 회음부 손상 정도의 차이는 독립 t 검정 및 일원분산분석을 이용하여 분석하였다.

## Results

### 연구 대상의 출산 실태와 회음손상 정도

자연주의 출산을 한 여성의 평균 연령은 33.18±3.68세이며 임신 전 BMI 평균은 20.94±2.82 kg/m^2^, 체중 증가는 평균 11.76±4.30 kg이었다. 초산부가 49.2%, 경산부가 50.8%였으며 39–40주 출산이 67.1%였다. 출산 경험자 중 이전 질식분만 경험자가 168명(92.3%)이었고 이전 제왕절개 대상자는 20명(11.0%)이었다. 전체 대상자 중 둘라 출산이 139명(38.8%)이고 수중실에서 진통한 경우가 103명(28.8%)이었으며 아기 출산 시의 자세는 반좌위가 156명(43.6%)이었고 그 다음으로 수중에서 앉아서, 옆으로 누워서, 앉아서, 엎드려서 순이었다. 출산한 아기는 남아가 53.6%이고 아기 머리둘레는 평균 34.46±1.19, 출생 시 체중은 평균 3.34 kg이었다(Table 1).

대상자 중 3명(0.8%)만 회음절개술을 받았으며 회음절개술을 받지 않은 대상자 중 1도 열상이 156명(43.6%), 2도 열상이 166명(46.4%)이었고 3도와 4도 열상은 8명(0.2%)과 1명(0.3%)이었다(Table 2). 분만1기 소요시간은 637.54±738.70분(약 10.6시간)으로 평균범위 내[21]에 있었으며, 분만2기 소요시간은 8.65±32.80분, 분만3기 소요시간은 19.78±15.85분이었다. 분만 동안 대상자의 35.5%가 1개 이상의 치료적 처치를 받았는데 항생제 투여가 39.7%, 수액 공급이 30.1%였다. 대상자의 99.7%는 출산 시 동반자가 남편이었으며 출생한 아기의 아프가 점수는 1분 평균 7.98±1.97, 5분 평균 8.99±0.09점이었다(Table 1).

### 연구 대상의 특성에 따른 회음부 손상 정도의 차이

본 연구에서 대상자의 일반적 특성에 따른 회음부 손상 정도는 대상자의 나이(F=9.15, *p*<.001)와 통계적으로 유의한 차이가 있었으며 체중 증가(F=1.62, *p*=.198), 임신 전 BMI (F=0.83, *p*=.476)와 분만 시 BMI (F=0.05, *p*=.995)는 통계적으로 유의한 차이가 없었다.

대상자의 산과적 특성에 따른 회음부 손상 정도는 출산력(t=19.13, *p*<.001), 경산부의 출산횟수(F=3.68, *p*=.027)와 이전 질식분만(F=3.00, *p*=.032), 아기 출산 시의 자세(F=7.44, *p*<.001), 치료적 처치 유무(t=–4.62, *p*<.001)와 통계적으로 유의한 차이가 있었다. 치료적 처치의 종류로는 수액 투여 유무(t=–2.72, *p*=.007), 산소 공급 유무(t=–2.76, *p*=.006), 경막외 마취 유무(t=–2.77, *p*=.006)와 통계적으로 유의한 차이가 있었다. 그러나 재태기간(F=1.72, *p*=.180), 둘라와 출산 유무(t=–0.27, *p*=.781), 진통 시 수중실 사용 유무(t=–0.19, *p*=.847), 회음절개술 유무(t=–0.55, *p*=.582) 및 치료적 처치 중에서는 항생제 사용 유무(t=–1.23, *p*=.222), 인공 양막 파열 유무(t=0.94, *p*=.346)와 회음부 손상 정도가 통계적으로 유의한 차이가 없었다.

또한 출생한 아기의 성별(t=–0.06, *p*=.947), 머리둘레(F=0.24, *p*=.864), 아기의 체중(F=1.95, *p*=.121)과 회음부 손상 정도는 통계적으로 유의하지 않았다(Table 1).

## Discussion

본 연구에서 대상자들은 의료적인 개입이 없는 자연주의 출산을 시도하였으며 출산 중 회음절개술이 필요한 대상자 3명을 제외하고는 대상자 358명에서 모두 예방적 회음절개술을 시행하지 않아 세계보건기구[22]와 미국산부인과 학회의 회음절개에 대한 권고사항을 잘 따르고 있었다.

먼저 산과적 특성을 살펴보면, 대상자의 50.8%가 경산부였으며 2번째 출산이 가장 많았지만 4번 이상의 출산도 0.9%나 되어 자연주의 출산을 선택하는 여성들이 다자녀를 선호하는 것으로 추정할 수 있었다. 경산부의 92.3%가 질식분만 경험이 있었으나 이전 분만이 자연주의 출산인지 구분되지 않아 산전 진찰의 부정적 느낌과 이전 자연주의 출산 경험이 자연주의 출산 선택의 영향요인임을 보고한 연구[13]와 비교할 수 없었다. 본 연구에서 자연주의 출산을 선택한 초산부도 49.2%로 상당한 비율을 보였다. 이는 TV 프로그램 및 유명인들의 자연주의 출산 경험이 소개되는 등[18] 자연주의 출산에 대해 정보가 보편화되고, 출산의 주체로 자기 결정권을 갖기 원하기 때문으로[13] 여겨진다. 또한 대상자들은 출산 동안 직접 남편의 지지를 받았는데 Lee와 Park [13]은 고립된 출산에서 아기와 남편까지 또 다른 주체자가 되는 점에서 고무적이며, 더 이상 낯선 출산방법이 아닌 자연주의 출산과 관련한 더 많은 연구가 필요함을 시사하였다.

본 연구에서 회음절개술을 시행하지 않은 대상자의 90%는 1도나 2도의 경증 회음부 열상이 있었지만, 3도 이상의 중증 회음부 열상 대상자는 2.5%였다. 이는 병원에서 의료인 중심으로 진행되는 질 분만(vaginal delivery) 대상자의 5%가 3도와 4도 회음부 열상인 것[17]과 비교하면 낮은 수치로, 자연주의 출산을 할 경우 중증 회음부 열상 비율이 적음을 의미한다. Baek [18]도 회음절개술을 하지 않아 생긴 열상은 깊지 않아 통증이 적고 회복기간이 짧다고 보고하면서 자연주의 출산 대상자들의 중증 회음부 열상의 비율이 낮은 것을 설명하고 있다.

본 연구에서 대상자의 회음부 손상 정도는 대상자의 나이와 관련이 있었다. 31–35세 대상자가 168명, 36–40세 대상자가 97명으로, 출산력, 경산부에서의 출산횟수, 이전 분만 경험과 연관시켜 볼 수 있다.

본 연구에서 초산부가 경산부보다 회음부 손상 점수가 높았고 경산부에서 이전 질식분만 경험이 없는 대상자가 이전 질식분만 경험이 있는 대상자보다 회음부 손상 점수가 높아 질식분만 경험을 포함한 출산력이 회음부 손상과 관계 있음을 확인하였다. 회음절개를 시행한 대만의 산부 대상 연구에서 Hsieh 등[20]도 초산부가 경산부보다 중증의 회음부 손상으로 고통을 겪으며 출산력이 회음부 손상의 강력한 예측인자임을 보고하였고, 메타분석 연구[19]도 초산부일수록 회음부 손상과 관련성이 높다고 보고하여, 출산력이 회음부 손상과 관련이 있는 것으로 파악되었다.

그러나, 이들 연구는 자연주의 출산 대상자에 대한 정확한 명시가 없어 본 연구의 결과와 비교하는 것이 제한적이다. 따라서 본 연구는 회음절개를 대부분 적용하지 않은 대상자의 회음부 손상 정도를 보고한 데 의의가 있다.

본 연구에서 회음부 손상은 1가지 이상의 치료적 처치를 받은 대상자와 처치가 불필요한 대상자와 관련이 있었다. 치료적 처치에서 경막외 마취, 산소 공급, 수액 투여와 회음부 손상이 관련이 있었는데 경막외 마취가 가장 높았으나 대상자가 18명으로 대상자의 수가 적어 타당성을 제시하기 어려웠다. Goueslard 등[23]과 Chia와 Huang [17]은 경막외 마취가 회음절개술의 위험을 더 높인다고 하였고 Garcia-Lausin 등[24]은 경막외 마취 대상자의 1.7%에서 심한 회음부 손상이 발생한다고 하였으나, 회음부 손상은 경막외 마취보다는 분만의 유형에 따라 달라지며[22] 경막외 마취가 심한 회음부 손상을 예방한다[25]는 연구가 있어 그 관련성을 명확히 설명할 수 없었다.

본 연구에서 아기 출산 시의 자세가 회음부 손상과 관련성이 있었는데, 좌위에서 회음부 손상 점수가 가장 높았고, 반좌위가 그 다음으로 회음부 손상 점수가 높았다. Hauck 등 [26]은 반좌위가 심한 회음부 손상을 줄이고 쇄석위가 심한 회음부 손상에 영향을 주었다고 했으며, Gupta 등[27]은 직립자세를 취한 여성이 똑바로 누운 자세를 취한 여성보다 2도 회음부 열상의 비율이 낮았다고 하여 출산시의 자세와 회음부 손상의 관련성이 있음을 보고하였다. 그러나 본 연구에서 좌위 자세로 아기를 출산한 경우는 13.1%에 불과하였고 산부의 선택, 돌보는 사람의 선호도, 의학적 처지에 따른 다양한 출산자세를 비교한 연구[27]도 대상자 수가 적어 본 연구결과와 비교하는 데는 무리가 있었다. 향후 연구를 통해 출산 시의 자세와 회음부 손상 정도를 파악하는 것이 필요하다고 생각된다. 또한 출산 중의 다양한 자세 변경이 권장되므로[27] 향후 연구는 이를 고려할 필요가 있다.

본 연구의 자연주의 출산 대상자 중 둘라와 출산한 대상자는 38.8%였다. 모든 대상자가 남편과 가족의 지지를 받고 있음에도 불구하고 분만을 도와주는 전문가를 필요로 한다는 것은 놀라운 일로, 둘라가 산모에게 전문적인 정보와 정서적 지지를 제공하여 출산을 이끌 수 있으며 둘라 출산을 한 여성은 만족감과 성취감이 높았다는 보고[9]와 일맥 상통한다. 본 연구에서 둘라 출산은 회음부 손상과 관련이 없었는데 이는 전체 자연주의 출산 대상자의 38.8%만이 둘라 출산을 하였기 때문으로 생각한다. 본 연구에서는 수중실에서 진통 과정을 진행한 대상자도 103명(28.8%)이었으며 이중 74명(71.8%) 수중에서 출산을 하였으나 진통 시 수중실 사용 여부에 따른 회음부 손상 정도에는 관련이 없었다. Gang과 Park [12]도 연구에서 수중실에서의 진통을 사용한 대상자는 31.6%였고, 이중 59.3%가 수중에서 출산을 하였으나 수중실에서 진통을 한 그룹과 수중실에서 진통을 하지 않은 그룹의 회음부 손상 정도에는 차이가 없다고 하여 본 연구 결과를 지지하였다.

본 연구를 통해 자연주의 출산 여성들의 출산 실태를 파악하여 자연주의 출산을 한 여성들은 회음절개술을 적용한 여성보다 경증의 회음부 손상이 있으며 이와 같은 손상은 크게 출산력과 연령이 관련 있음을 확인하였다. 그러나 본 연구에서 출산한 아기의 상태와 회음부 손상과는 관련이 없어 Hauck 등[6]이 아기의 출생 시 체중과 태향이 회음부 손상과 관련 있다고 한 연구 결과와 달랐다. 이는 산모의 출산 시 자세나 아기의 상태, 출산환경과 관련이 있을 것으로 추정해볼 수 있으며 향후 연구에서 확인하여야 할 것이다.

또한 본 연구는 일개 병원에서 자연주의 출산을 한 여성들의 출산 의무기록을 분석한 후향적 연구로 자연주의 출산 여성 모두의 결과로 일반화하기 어렵고, 대상자들의 분만과정에서 생기는 문제 개선을 위한 중재방안을 모색하는 데 한계가 있었다. 그러나 저출산 시대임에도 358명 자연주의 출산 대상자의 출산 실태를 파악하였다는 데 연구의 의의가 있으며, 향후 자연주의 출산을 시행하는 다양한 형태의 기관에서의 연구를 위한 기초자료를 제시하였다.

결론으로, 본 연구는 자연주의 출산을 한 여성 대상의 출산 실태에 대한 후향적 조사를 통해 회음부 손상이 대상자의 나이, 출산력, 이전 질식분만 경험, 아기 출산 시의 자세, 치료적 처치와 관련 있음을 확인하였다. 또한 자연주의 출산 여성의 39%가 둘라를 이용하였으나 회음부 손상과 관련이 없었고 선행연구에서 유의한 변수로 제시된 아기 머리둘레, 체중도 유의미하지 않았다. 따라서 향후 자연주의 출산과 회음부 손상 영향요인과의 관련성을 확인하는 연구가 필요하며, 출산자세, 수중분만, 둘라와 출산 동반자의 기대효과와 분만 만족도를 확인하는 전향적 연구가 필요함을 시사한다.

## Figures and Tables

**Table 1.** Differences in degree of perineal injury in women with natural childbirth (N=358)

Variable	Categories	n (%)	Degree of perineal injury, mean±SD	t/F	*p*
*General characteristics*					
Age (year)	≤25	8 (2.2)	1.50±1.06	9.15	<.001
	26–30	79 (22.1)	1.82±0.59		
	31–35	168 (46.9)	1.38±0.65		
	36–40	97 (27.1)	1.31±0.61		
	≥41	6 (1.7)	1.0±0.00		
Weight gain (kg)	<11.5	169 (47.2)	1.43±0.65	1.62	.198
	11.5–16	144 (40.2)	1.44±0.69		
	>16	45 (12.6)	1.62±0.61		
Pre-pregnancy BMI (kg/m^2^)	<18.5	53 (14.8)	1.49±0.60	0.83	.476
	18.5–22.9	240 (67.0)	1.45±0.68		
	23.0–24.9	38 (10.6)	1.55±0.64		
	≥25.0	27 (7.6)	130±0.60		
BMI at delivery (kg/m^2^)	<18.5	0 (0.0)		0.05	.995
	18.5–22.9	82 (22.9)	1.45±0.63		
	23.0–24.9	102 (28.5)	1.46±0.67		
	≥25.0	174 (48.6)	1.45±0.68		
*Obstetric characteristics*					
Parity	Primiparity	176 (49.2)	1.94±0.50	19.13	<.001
	Multiparity	182 (50.8)	0.99±0.43		
Number of births in multiparity	2	141 (38.4)	0.99±0.36	3.68	.027
	3	38 (10.6)	1.03±0.59		
	4 or more	3 (1.2	0.33±0.57		
Gestational period (week)	37–38	68 (19.0)	1.32±0.65	1.72	.180
	39–40	240 (67.1)	1.48±0.66		
	≥41	50 (13.9)	1.52±0.67		
Previous vaginal delivery in multiparity	None	14 (7.7)	1.14±0.36	3.00	.032
	1	135 (74.2)	0.99±0.37		
	2	30 (16.5)	97±0.61		
	3 or more	3 (1.6)	0.33±0.57		
Previous cesarean section in multiparity	Yes	20 (11.0)	1.15±0.36	–2.03	.052
	No	162 (89.0)	0.97±0.43		
Birth with doula	Yes	139 (38.8)	1.47±0.62	–0.27	.781
	No	219 (61.2)	1.45±0.69		
Water room use in labor	Yes	103 (28.8)	1.47±0.69	–0.19	.847
	No	259 (71.2)	1.45±0.65		
Episiotomy	Yes	3 (0.8)	1.67±0.57	–0.55	.582
	No	355 (99.2)	1.45±0.66		
Birthing posture	Semi-fowlers	156 (43.6)	1.52±0.66	7.44	<.001
	Sitting position in the water	74 (20.7)	1.31±0.70		
	Side-lying	61 (17.0)	1.26±0.57		
	Sitting	47 (13.1)	1.83±0.56		
	Hand knee	20 (5.5)	1.20±0.61		
Therapeutic procedures	Yes	127 (35.5)	1.67±0.65	–4.62	<.001
	No	231 (64.5)	1.34±0.64		
Therapeutic procedure type for ‘yes’ response (n=127)	Antibiotic			–1.23	.222
	Yes	83 (65.4)	1.72±0.63		
	No	44 (34.6)	1.57±0.69		
	Hydration			–2.72	.007
	Yes	63 (49.6)	1.83±0.66		
	No	64 (50.4)	1.52±0.61		
	Oxygen			–2.76	.006
	Yes	28 (22.0)	1.96±0.63		
	No	99 (78.0)	1.59±0.63		
	Epidural anesthesia			–2.77	.006
	Yes				
	No	18 (14.2)	2.06±0.53		
	AROM	109 (85.8)	1.61±0.65		
	Yes			0.94	.346
	No	17 (13.4)	1.53±0.94		
		110 (86.6)	1.69±0.60		
Baby’s sex	Male	192 (53.6)	1.45±0.62	–0.06	.947
	Female	166 (46.4)	1.46±0.71		
Baby head circumference^†^ (cm)	<32.5	13 (3.6)	1.46±0.77	0.24	.864
	32.5–34.3	163 (45.5)	1.44±0.62		
	34.3–36.3	165 (46.1)	1.45±0.70		
	≥36.4	17 (4.7)	1.58±0.71		
Baby body weight^†^ (g)	<2,750	14 (3.9)	1.57±0.51	1.95	.121
	2,750–3,340	163 (45.5)	1.53±0.64		
	3,350–4,140	173 (48.3)	1.39±0.70		
	≥4,150	8 (2.2)	1.13±0.35		

AROM: Artificial rupture of membrane; BMI: body mass index.

† Baby head circumference and body weight were categorized according to less than 10%, 10%–50%, 50%–90%, over 90%.

**Table 2.** Degree of perineal damage in women with natural childbirth (N=358)

Variable	Categories	n (%)	Mean±SD
Degree of perineal injury	Episiotomy (degree)	3 (0.8)	1.67±0.57
	1	1 (0.3)	
	2	2 (0.5)	
	No episiotomy (degree)	355 (99.2)	1.45±0.66
	Intact	24 (6.7)	
	1	156 (43.6)	
	2	166 (46.4)	
	3	8 (2.2)	
	4	1 (0.3)	
Labor time (minute)	Stage I ^‡^		637.54±738.70
	Stage II ^‡^		8.65±32.80
	Stage III ^‡^		19.78±15.85
Type of therapeutic procedure (n=209^†^)	Antibiotics	83 (39.7)	
	Hydration	63 (30.1)	
	Oxygen	28 (13.4)	
	Epidural anesthesia	18 (8.6)	
	Artificial rupture of membrane	17 (8.1)	
Birth companion	Husband	357 (99.7)	
	Mother	1 (0.3)	
Apgar score	1 minute		7.98±1.97
	6	3 (0.8)	
	7	1 (0.3)	
	8	353 (98.6)	
	9	1 (0.3)	
	5 minutes		8.99±0.09
	8	3 (0.8)	
	9	355 (99.0)	

† Number of duplicate responses: including cases where one person received more than one.

‡ Stage I: true labor to full cervical dilatation; stage II: full cervical dilatation to childbirth; stage III: childbirth to placenta birth.
